# HLA-DRB1*14 is associated with lower odds of symptomatic SARS-CoV-2 infection in Mexican individuals

**DOI:** 10.3389/fimmu.2026.1907153

**Published:** 2026-09-01

**Authors:** Eric G. Hernández, Diego A. Daza-Mendoza, Enrique González-Rivas, Angélica Serrano-Vázquez, Horacio Pérez-Juárez, Alina Uribe-García, Gareth O. Rostro-Alonso, Patricia Morán-Silva, Liliana Rojas-Velázquez, Cecilia Ximénez García

**Affiliations:** 1 Laboratorio de Inmunología, Unidad de Medicina Experimental “Doctor Ruy Pérez-Tamayo”, Facultad de Medicina, Universidad Nacional Autónoma de México (UNAM), Ciudad de México, Mexico; 2 Carrera de Biología, Facultad de Estudios Superiores (FES)-Iztacala, Estado de México, Mexico; 3 Laboratorio de Farmacogenética, Unidad Multidisciplinaria de Investigación Experimental Zaragoza (UMIEZ), Facultad de Estudios Superiores (FES)-Zaragoza, Ciudad de México, Mexico

**Keywords:** COVID-19, *HLA-DRB1*, Mexican population, next generation sequence, SARS-CoV-2 infection

## Abstract

Mexico has been one of the countries most affected by the COVID-19 pandemic. Several factors can influence the susceptibility to SARS-CoV-2 infection, particularly highly polymorphic genes in the HLA system. Therefore, this pilot study analyzed the association between HLA-DRB1 genetic polymorphisms and the susceptibility and/or resistance to symptomatic SARS-CoV-2 infection in a population from the Metropolitan Area of the Valley of Mexico. Individuals with a confirmed SARS-CoV-2 infection were recruited (n = 90), as well as a seronegative control group without reported symptomatic infection (n = 60). The HLA-DRB1 locus was typed using NGS. We found a significant increase in the frequency of the HLA-DRB1*14 allele in the control group (OR = 0.37; 95% CI: 0.18–0.72; p = 0.003; pc = 0.03). These data suggest that HLA-DRB1*14, a prevalent allele in the Mexican population, is associated with a reduced risk of symptomatic SARS-CoV-2 infection in this cohort.

## 1 Introduction

The COVID-19 global pandemic caused by the SARS-CoV-2 virus, left numerous damages around the world. The virus caused a pandemic that triggered a global crisis, with more than 7 million deaths reported worldwide to date. Mexico is among the most affected countries, ranking the fifth most deaths at nearly 335,000, behind the US, Brazil, India, and Russia, according to documents published by the World Health Organization (1).

Mexico City, the nation’s capital, recorded 264,330 infections, and the State of Mexico, in the central region of the country and bordering Mexico City, reported 125,628 infections (2). Both states constitute the Metropolitan Area of the Valley of Mexico (MAVM) (**Supplementary Figure 1**) and are considered the national epicenter of the pandemic because of their close and solid labor, academic, commercial, and economic relationships (3, 4). It has been shown that local factors such as overcrowded areas, frequent social interactions, and full public transport can increase the number of infections by SARS-CoV-2 due its mechanism of transmission; the virus can persist in aerosols and on surfaces (5, 6). It is a large and densely populated region with a genetically heterogeneous population, is connected through a dense public transport network to Mexico City and is the area that was most exposed to the COVID-19 health emergency (3).

Although the differences in infection susceptibility and severity between populations are difficult to explain, genetic and environmental factors play crucial roles in viral pathogenesis. A variety of studies are being conducted on human genetic elements that may contribute to the observed diversified disease severity to better understand how the clinical course may be influenced by the patient’s genetics, and to identify which aspects could be related to SARS-COV-2 clinical variability (7, 8). Adaptive immune response is a critical component of protection against viral pathogens. CD4 T cells promote virus-specific antibodies by acting on T-dependent B cells (9). Classical HLA class II molecules (HLA-DR, HLA-DQ, and HLA-DP) are heterodimers composed of alpha and beta chains encoded by genes within the MHC and can present peptides from proteins that have been taken up from outside the cell via endocytosis to CD4+ helper T lymphocytes (10, 11). The expression of class II molecules is limited to cells with specialized immune functions, collectively known as professional antigen-presenting cells, primarily dendritic cells, macrophages, and mature B lymphocytes. In addition, HLA class II molecules are highly expressed on the surface of epithelial cells in both the lungs and intestine (12), which is potentially relevant in the context of SARS-CoV-2 infection (13). Reports have described the effectiveness of presenting SARS-CoV-2 structural viral proteins through HLA class II molecules (11).

For this study, we investigated whether individuals symptomatic to SARS-CoV-2 infection have different *HLA-DRB1* alleles compared to seronegative controls without reported symptomatic infection in this population of the MAVM.

## 2 Materials and methods

### 2.1 Study participants and recruitment

As part of an ethically approved research study “Genetic polymorphism of the HLA (*Human Leukocyte Antigen*) system in COVID-19 patients from Mexico City”, the study protocol was evaluated and approved by the ethics and research committee of the Faculty of Medicine of the National Autonomous University of Mexico (FM/DI/059/2023). Participation was voluntary, and specific procedures were presented in writing for informed consent. All participants were over 18 years old and provided their permission. We compared *HLA-DRB1* allele frequencies in two groups: symptomatic patients who developed SARS-CoV-2 infection and individuals who remained without reported symptomatic infection following exposure to SARS-CoV-2. The data collected from all the participants were captured in electronic databases, anonymized, and managed confidentially.

### 2.2 Symptomatic individuals’ group

Volunteers were recruited between August 2023 and September 2024. Adult patients residing in MAVM were enrolled. Genetically unrelated patients met the following criteria: 1) positive clinical diagnosis of SARS-CoV-2 by *RT-PCR*; 2) seropositivity for SARS-CoV-2; 3) symptoms due to COVID-19; 4) no symptoms related to other respiratory diseases; and 5) no autoimmune diseases.

During recruitment, we received responses from symptomatic individuals who tested positive for SARS-CoV-2 antibodies during the pandemic, expressed their willingness to participate, and provided samples for DNA analysis.

### 2.3 Control group of seronegative individuals with no reported symptomatic infection

A group of seronegative individuals was also formed, with no report of symptomatic infection during the pandemic (data obtained for self-report, from a questionnaire completed prior to agreeing to participate in the study), who met the same demographic characteristics, in addition to the following criteria: 1) no history of infection and/or symptoms during the COVID-19 pandemic, 2) seronegative to SARS-CoV-2 (titers less than 0.520 OD, ELISA), 3) no symptoms of COVID-19 at the time of sample collection, and 4) no autoimmune diseases.

On the day of sample collection, all participants were interviewed to obtain relevant clinical data such as sex, age, weight, height, comorbidities, and COVID-19 history.

### 2.4 DNA sample collection and processing

A sample of 5 ml of whole blood was drawn from each patient and the control group. Genomic DNA was isolated from peripheral EDTA-anticoagulated whole blood using a modified *salting-out* microtechnique (14). The samples were centrifuged to separate the leukocyte components (approximately 1 ml with 2 × 106 cells/ml) from the plasma. Erythrocytes were eliminated from the leukocyte fraction by re-suspension in a lysis buffer solution composed of sucrose, Tris-HCl 2M, MgCl2, and Triton 100%. Next, leukocytes were lysed with a second lysis buffer solution composed of NaCl and EDTA. The DNA was separated from the proteins with SDS, NaClO4, and NaCl; precipitated with isopropanol at -20 °C; and maintained at that temperature for 12 h. DNA was next washed three times with 70% ethanol at -20 °C. The ethanol was then decanted, and the DNA was dried at room temperature and rehydrated in sterile distilled water. When DNA was quantified for spectrophotometry, the average concentration obtained was 250 ng/μl in 300 μl volume, and its integrity was tested by electrophoresis in 0.8% agarose gels. HLA-DRB1 genotyping was performed by next-generation sequencing (NGS). Owing to the slow process of collecting samples from patients and controls, DNA extraction was performed at the time of sample acquisition, thus avoiding the accumulation of samples and the possibility of cross-contamination.

### 2.5 HLA-DRB1 typing by NGS sequencing

High-resolution HLA-DRB1 typing tests were developed and their performance characteristics were determined by CD Genomics Inc (NY, USA). We sent 150 samples for NGS according to the company’s standard protocols. Sequencing was performed using the PacBio’s Single Molecule Real-Time (SMRT) platform, and the resulting data were analyzed using specialized bioinformatics techniques to identify allelic variants.

The methodology for typing HLA-DRB1 alleles is described in detail below.

#### 2.5.1 Reference database and version

The allele reference database used for HLA allele assignment was the IPD-IMGT/HLA Database: https://www.ebi.ac.uk/ipd/imgt/hla/.

The version used for this analysis was Release 3.59.0 (January 2025).

#### 2.5.2 Software used

Alleles were assigned using BLAST (Basic Local Alignment Search Tool). The BLAST+ executable package is available at the NCBI (https://ftp.ncbi.nlm.nih.gov/blast/executables/blast+/).

#### 2.5.3 Quality control criteria

A comprehensive set of quality control (QC) parameters was used throughout the analysis. The primary QC criteria are summarized below.

1. Minimum read depth per allele across the target region: ≥100×
2. Minimum base quality: QV20
3. Allele support thresholds:

Homozygous calls: ≥ 95%. For a homozygous call, the predominant nucleotide at each position was supported by at least 95% of the sequencing reads. Heterozygous calls: ≥ 80%. For a heterozygous call, the predominant nucleotide of each allele at each position must be supported by at least 80% of the sequencing reads assigned to that allele.

Allele calls that did not meet these QC criteria were flagged for manual review, and if necessary, for repeat testing.

#### 2.5.4 Ambiguity handling

For these samples, HLA typing was performed by sequencing the complete HLA-DRB1 antigen recognition site (ARS), corresponding to exon 2. Our PCR primers were designed to anneal within introns 1 and 2, thereby allowing complete coverage of exon 2 within a single continuous amplicon. Cis-/transphasing ambiguities did not occur within the sequenced region.

For ambiguities resulting from nucleotide variations located outside exon 2, where the exon 2 sequences remained completely identical, the results were consolidated and reported as G-group codes. This report follows the guidelines of the IPD-IMGT/HLA Nomenclature and IPD-IMGT/HLA Database Release 3.59.0 (published in January 2025).

### 2.6 Statistical analysis

Allele frequency was calculated using direct counting. The statistical associations between the allelic frequencies of the *HLA-DRB1* locus and the comorbidities present with the clinical manifestations of the disease were analyzed using 2 × 2 contingency tables and Fisher’s exact test. Statistical significance was set than 0.05. If appropriate, p-values were corrected using the Bonferroni method (for allele frequencies, multiplying the original p-value by the number of alleles identified within the group). Odds ratios and 95% confidence intervals (95% CI) were calculated for risk estimation. Statistical software Epi-info (version 6; Centers for Disease Control and Prevention, Atlanta, GA, USA) was used to perform these estimations. However, logistic regression analyses were conducted at the individual level, coding each participant according to carrier status for each HLA-DRB1 allele (carrier vs. non-carrier), to estimate the association between allele carriage and the risk of symptomatic SARS-CoV-2 infection. When associations between HLA alleles and symptomatic SARS-CoV-2 infection were statistically significant using 2 × 2 contingency tables, multivariate logistic regression analysis was performed to adjust for potential confounding factors. This analysis was performed using Jamovi software, version 2.4.8 (15).

## 3 Results

In total, 150 individuals were recruited from different MAVM municipalities and boroughs (**Supplementary Figure 2**). Details of the symptomatic and control individuals are shown in **Table 1**.

**Table 1:** Demographic data of symptomatic and controls individuals.

Variable	Symptomatics (n=90)	Controls (n=60)	*p-value*
Male sex, n (%)	39 (43.4%)	19 (31.6%)	*0.16*
Age (years)	27 (21.0-45.5)	23 (19.0-40.2)	*0.06*
BMI (kg/m^2^)	25.31 (22.8-27.3)	24.88 (22.4-28.0)	*0.71*

Data are presented as median (interquartile range) or number (percentage). P-values were calculated using the chi-square test for categorical variables and the Mann–Whitney U test for continuous variables.

All p-values were greater than 0.05 between the two groups in terms of age, body mass index (BMI), and sex, according to the chi-square and Mann–Whitney U tests (p = 0.05). Advanced age did not show a statistically significant association.

The most frequent comorbidities were obesity (13 in the symptomatic group vs. 5 in the asymptomatic group), smoking (11 vs. 7), diabetes (4 symptomatic), chronic digestive disease (4 symptomatic vs. 4 asymptomatic), and hypertension (4 symptomatic vs. 1 asymptomatic), with a higher prevalence in the symptomatic group. However, none of these differences were statistically significant according to the chi-square test. Data corresponding to the main comorbidities are shown in **Table 2**.

**Table 2:** Clinical characteristics of symptomatic and controls individuals.

	Symptomatic	Controls			*p value*
Elderly people (>60 years old) n (%)	7 (7.77%)		3 (5%)	0.53	
Comorbidities n (%)					
Obesity	13 (14.44%)		5 (8.33%)	0.28	
Diabetes	4 (4.44%)		0 (0%)	0.10	
Chronic digestive diseases	4 (4.44%)		4 (6.66%)	0.53	
Smokers	11 (12.22%)		7 (11.66%)	0.91	
Hypertension	4 (4.44%)		1 (1.66%)	0.36	
Depression	1 (1.11%)		3 (5%)	0.15	
Insulin resistance	0 (0%)		2 (3.33%)	0.13	
Surgeries	1 (1.11%)		1 (1.66%)	0.81	
Hypothyroidism	2 (2.22%)		2 (3.33)	0.66	
Pulmonary fibrosis	1 (1.11%)		0 (0%)	0.36	
Prediabetes	1 (1.11%)		0 (0%)	0.36	
Fatty liver	0 (0%)		1 (1.66%)	0.36	

Given the low prevalence of comorbidities, Fisher’s exact test was used when the expected cell count was < 5.

### 3.1 Allelic frequencies

The *HLA-DRB1* locus was typed in the symptomatic and control MAVM groups (**Supplementary Table 1**). In total, 38 different alleles were detected in the 150 individuals, 27 of which had very low frequencies. The most common alleles were HLA-DRB1*08:02 (AF = 0.18), HLA-DRB1*04:07 (AF = 0.1), and HLA-DRB1*14:06 (AF = 0.08).

In the symptomatic group, we detected 16 alleles in the HLA-DRB1*14 group in 15 individuals with one homozygous person, whereas in the control group, we detected 25 alleles in 23 individuals with two homozygous individuals.

These findings are consistent with those previously reported by various authors, who documented that these alleles are highly prevalent in the Mexican population (16–19) (**Supplementary Table 2**).

Because multiple alleles had very low frequencies but could be grouped according to the first field of HLA nomenclature (e.g., HLA-DRB1*14), the analysis was performed at two levels: first by allele specificity, to determine if any variant was associated with the disease (**Table 3**). Correction at the allele-specific level was performed with 11 comparisons, and subsequently by group of alleles, to identify general associations and increase the combined frequency. Correction at the allele group level was performed with 10 comparisons (thus improving statistical power) **Table 4**.

**Table 3:** Comparison of the *HLA-DRB1* alleles frequencies between symptomatic and control groups of the MAVM.

	Symptomatic N= 90		Control N= 60			
HLA-DRB1*	n	a.f.	n	a.f.	*p*	OR (CI 95%)
*08:02*	26	0.144	30	0.25	0.0215	0.5 (0.28-0.91)
*04:07*	16	0.08	14	0.11	0.43	0.73 (0.34-1.57)
*14:06*	10	0.055	14	0.116	0.055	0.44 (0.19-1.03)
*07:01*	13	0.072	6	0.05	0.43	1.47 (0.54-4)
*04:04*	12	0.066	4	0.033	0.2	2.07 (0.65-6.58)
*16:02*	12	0.066	4	0.033	0.29	2.07 (0.65-6.58)
*14:02*	5	0.027	9	0.075	0.09	0.35 (0.11-1.07)
*15:01*	9	0.05	5	0.041	0.73	1.21 (0.39-3.70)
*04:11*	6	0.033	6	0.05	0.47	0.65 (0.20-2.08)
*03:01*	9	0.05	1	0.008	0.054	6.26 (0.78-50.09)
*11:01*	6	0.033	3	0.025	0.67	1.34 (0.32-5.48)

N, Number of samples; n, Number of alleles; a.f, Allele Frequency; OR, Odds ratio; CI, Confidence interval; *p*, P value.

**Table 4:** *HLA-DRB1* allele groups and their association with symptomatic SARS-CoV-2 infection in the population of the MAVM.

	Symptomatic N= 90		Control N= 60				
HLA-DRB1* Allele-family (n)	n	a.f.	n	a.f.	*p*	OR (CI 95%)	*pc*
*14* (41)	16	0.08	25	0.20	**0.003**	0.37 (0.18-0.72)	**0.03**
*08* (61)	31	0.17	30	0.25	0.1	0.62 (0.35-1.09)	1
*04* (75)	45	0.25	30	0.25	1	1 (0.58-1.70)	1
*07* (19)	13	0.072	6	0.05	0.43	1.47 (0.54-4)	1
*01* (15)	12	0.066	3	0.025	0.1	2.78 (0.76-10.08)	1
*16* (18)	15	0.077	4	0.033	0.09	2.63 (0.85-8.14)	0.9
*15* (18)	12	0.061	7	0.058	0.77	1.15 (0.44-3.01)	1
*13* (17)	10	0.055	7	0.058	0.92	0.94 (0.35-2.56)	1
*11* (16)	11	0.061	5	0.041	0.6	1.49 (0.50-4.42)	1
*03* (11)	10	0.055	1	0.008	0.054	7 (0.88-55.41)	0.54

N, Number of samples; n, Number of alleles, a.f, Allele Frequency; OR, Odds ratio; CI, Confidence interval; p, P value; pc, P corrected.

Analysis using 2 × 2 contingency tables between symptomatic and control individuals showed statistically significant differences in the allele frequencies of the HLA-DRB1*08:02 allele (OR = 0.5; 95% CI: 0.28–0.91; p = 0.0215) and the HLA-DRB1*14 allele group (OR = 0.37; 95% CI: 0.18–0.72; p = 0.003). Both increased in frequency in the control individuals (25% and 20% compared with 14% and 8% in symptomatic individuals, respectively), which could be associated with a lower probability of symptomatic infection. However, after applying the Bonferroni correction, only the HLA-DRB1*14 allele maintained significance (*pc* = 0.03), whereas HLA-DRB1*08:02 lost significance (*pc* = 0.23). However, the latter showed a trend that could reach significance with a larger sample size.

### 3.2 Logistic regression

Subsequently, a multivariate logistic regression adjusted for age, sex, BMI, and the presence of comorbidities was performed, in which HLA-DRB1*14 showed a statistically significant association with a lower risk of developing a symptomatic SARS-CoV-2 infection (OR = 0.35; 95% CI: 0.16–0.76; p = 0.008) (**Supplementary Table 3**). This finding suggests that carriers of HLA-DRB1*14 had approximately 65% lower adjusted odds of symptomatic SARS-CoV-2 infection than non-carriers.

## 4 Discussion

Since the beginning of the global COVID-19 pandemic, intensive efforts have been made to deduce the genetic and immune features that may underlie variations in susceptibility to SARS-CoV-2 infection as well as disease outcomes in COVID-19 (13).

During the pandemic, we did not have access to patients with varying degrees of the disease; therefore, we opted to conduct a retrospective case-control study and invited our population to participate freely and voluntarily with all the information that interested individuals required.

Notably, this preliminary result is part of a larger study involving complete HLA haplotype typing of the participating population; however, we consider it relevant to report this first finding in the *HLA-DRB1* locus of our population.

Something potentially relevant to justify our research at this locus is the expression of HLA class II molecules in lung and intestinal epithelial cells, as well as the contributions of Weingarten-Gabbay and collaborators who used two lung epithelial cell lines that mainly expressed HLA-DR heterodimers and less so to HLA-DP and HLA-DQ heterodimers in both cell lines: A549 and HEK293T (20, 21), with the expression of *HLA-DRB* alleles highly prevalent in European (EUR) and US populations, including HLA-DRB1*07:01 (~14% EUR, ~12% US), HLA-DRB1*11:04, -DRB4*01:01, -DRB1*15:01 expressed by A549 and HLA-DRB1*15:01 (~14% EUR, ~11% US), and HLA-DRB5*01:01 (~16% EUR), expressed by HEK293T. These studies have mainly described the presentation of SARS-CoV-2 structural protein antigens through HLA class II complexes (22).

Regarding the associations of the *HLA-DRB1* locus with the symptomatic infection, in the present study we found that the HLA-DRB1*14 allele group appears to be associated with a lower risk of developing a symptomatic SARS-CoV-2 infection. This finding partially agrees with that reported in an Iranian population study (23), where a similar trend toward a protective effect was observed, although this association was not statistically significant after correction for multiple testing. Similarly, Akcay et al. (24) reported an association between this allele and an increased susceptibility to infection in Turkish kidney transplant recipients, a group characterized by chronic immunosuppression. These discrepancies can be attributed to both the clinical context (as the immunological conditions of immunocompromised individuals differ from those of the general population) and genetic variability among populations.

In contrast, symptomatic individuals exhibited a lower frequency of HLA-DRB1*08:02 infection than control individuals. Unfortunately, after applying the Bonferroni correction to this result, the statistical significance was lost.

In this study, although advanced age and comorbidities were more frequent in the symptomatic group than in the seronegative control group without reported symptomatic infections, none of these variables showed a statistically significant association with SARS-CoV-2 infection. It is well known that advanced age, diabetes, hypertension, coronary artery disease, COPD, and autoimmune diseases increase the risk of developing severe forms of COVID-19 after the SARS-CoV-2 infection (25–27), with advanced age being one of the main risk factors due to the deterioration and dysregulation of the immune system (28). In this context, our results could be explained by the demographic characteristics of the analyzed cohort, composed mostly of young individuals with a mean age close to 30 years and with a low representation of older people (only 7 elderly individuals among the patients and 3 among the controls), as well as by a low frequency of comorbidities, probably for the same reason.

It is important to highlight the numerous discrepancies among studies on the association between HLA and SARS-CoV-2 infections. This can be attributed to multiple factors such as the genetic context of the populations studied, limited sample sizes that reduce statistical power, and differences in the definition of the disease phenotypes (13). Furthermore, the timing of many of these studies (during an active health emergency) limited access to patients and controls with similar clinical characteristics, and introduced variables that are difficult to control, such as vaccination status or the circulation of different viral variants.

Our work adds to a growing list of studies that have found an association between HLA class II alleles, which could be associated with conferring protection against SARS-CoV-2 infection in populations from different regions of the world. These included alleles, such as HLA-DRB1*03:01 in a population from Sardinia, Italy (25). In a cohort of 69 asymptomatic and 78 symptomatic individuals in the United Kingdom, the HLA-DRB1*04:01 allele was found to be more frequent in the asymptomatic group (29, 30). Furthermore, Abolnezhadian et al. (31) reported that the HLA-DRB1*11:05, -DRB1*14:01, -DRB1*13:05, and -DRB1*04:01 alleles were involved in resistance to SARS-CoV-2 infection in a group of 109 infected patients and 70 healthy controls in an Iranian population. These authors also described protective associations against SARS-CoV-2 infection at class I HLA loci.

Our study is limited by its relatively small sample size. However, our results suggest that HLA-DRB1*14 alleles may be associated with a reduced risk of symptomatic SARS-CoV-2 infection, this allele was significantly overrepresented in our control group (20%) compared to symptomatic (8%) individuals. This result needs to be confirmed in the future with an increased number of symptomatic and seronegative controls without symptomatic MAVM-infected individuals. Another important limitation of our study is that the absence of all symptoms in the control group was self-reported; vaccination data, such as vaccine type, number of doses, dates, and timing relative to infection, were unavailable; ancestry analysis, limited power for rare alleles, lack of external replication, and the absence of functional validation were also not performed.

It is also important to mention that in addition to increasing the sample size of our study population, the remaining HLA class I (A, B, and C) and the two remaining HLA class II (DP and DQ) haplotypes should be typed to explore the possibility of finding haplotypes that could confer protection against symptomatic SARS-CoV-2 infection and predict SARS-CoV-2 peptide binding to HLA-DRB1*14.

Further studies in the Mexican population are necessary to determine the differences in some of the wealthiest admixed populations and to understand the influence of genetic background in responding to diseases that have markedly differed around the country.

Finally, the evidence shown by different studies in populations worldwide during and after the COVID-19 pandemic demonstrates that the prevalent HLA alleles and haplotypes in affected populations are efficient presenters of SARS-CoV-2 antigens to generate an efficient adaptive immune response.

## Data Availability

The original contributions presented in the study are included in the article/**Supplementary Material**. Further inquiries can be directed to the corresponding author.
