# Supplementary material for: HLA-DRB1*14 is associated with lower odds of symptomatic SARS-CoV-2 infection in Mexican individuals
Source: Front Immunol. 2026 Sep 1;17:1907153. doi: 10.3389/fimmu.2026.1907153 (PMC13574783; doi:10.3389/fimmu.2026.1907153)
Supplement: Supplementary file 1 [file DataSheet1.pdf]

**Supplementary Table S1. Allele counts in each group.**

| Allele-Specific | Controls | | Symptomatic | |
|---|---|---|---|---|
| | n | a.f | n | a.f |
| **08:02** | 30 | 0.25 | 26 | 0.14444444 |
| **01:01** | 1 | 0.00833333 | 5 | 0.02777778 |
| **04:04** | 4 | 0.03333333 | 12 | 0.06666667 |
| **14:02** | 9 | 0.075 | 5 | 0.02777778 |
| **08:01** | 0 | 0 | 3 | 0.01666667 |
| **03:01** | 1 | 0.00833333 | 9 | 0.05 |
| **04:07** | 14 | 0.11666667 | 16 | 0.08888889 |
| **11:04** | 2 | 0.01666667 | 5 | 0.02777778 |
| **04:11** | 6 | 0.05 | 6 | 0.03333333 |
| **11:01** | 3 | 0.025 | 6 | 0.03333333 |
| **04:10** | 2 | 0.01666667 | 1 | 0.00555556 |
| **03:02** | 0 | 0 | 1 | 0.00555556 |
| **04:03** | 1 | 0.00833333 | 3 | 0.01666667 |
| **01:02** | 2 | 0.01666667 | 5 | 0.02777778 |
| **07:01** | 6 | 0.05 | 13 | 0.07222222 |
| **13:01** | 3 | 0.025 | 3 | 0.01666667 |
| **04:05** | 2 | 0.01666667 | 2 | 0.01111111 |
| **08:04** | 0 | 0 | 2 | 0.01111111 |
| **14:06** | 14 | 0.11666667 | 10 | 0.05555556 |
| **15:03** | 1 | 0.00833333 | 0 | 0 |
| **04:08** | 1 | 0.00833333 | 0 | 0 |
| **13:05** | 1 | 0.00833333 | 2 | 0.01111111 |
| **04:01** | 0 | 0 | 2 | 0.01111111 |
| **15:01** | 5 | 0.04166667 | 10 | 0.05555556 |
| **01:03** | 0 | 0 | 2 | 0.01111111 |
| **16:02** | 4 | 0.03333333 | 13 | 0.07222222 |
| **13:03** | 2 | 0.01666667 | 2 | 0.01111111 |
| **09:01** | 0 | 0 | 3 | 0.01666667 |
| **15:02** | 1 | 0.00833333 | 2 | 0.01111111 |
| **04:42** | 0 | 0 | 1 | 0.00555556 |
| **04:02** | 0 | 0 | 2 | 0.01111111 |
| **13:04** | 0 | 0 | 1 | 0.00555556 |
| **14:01** | 1 | 0.00833333 | 1 | 0.00555556 |
| **10:01** | 2 | 0.01666667 | 1 | 0.00555556 |
| **13:02** | 1 | 0.00833333 | 2 | 0.01111111 |
| **16:01** | 0 | 0 | 2 | 0.01111111 |
| **12:01** | 0 | 0 | 1 | 0.00555556 |
| **14:05** | 1 | 0.00833333 | 0 | 0 |

| Allele-Group | Controls | | Symptomatic | |
|---|---|---|---|---|
| | n | a.f | n | a.f |
| **08** | 30 | 0.25 | 31 | 0.17222222 |
| **01** | 3 | 0.025 | 12 | 0.06666667 |
| **04** | 30 | 0.25 | 45 | 0.25 |
| **14** | 25 | 0.20833333 | 16 | 0.08888889 |
| **03** | 1 | 0.00833333 | 10 | 0.05555556 |
| **11** | 5 | 0.04166667 | 11 | 0.06111111 |
| **07** | 6 | 0.05 | 13 | 0.07222222 |
| **13** | 7 | 0.05833333 | 10 | 0.05555556 |
| **15** | 7 | 0.05833333 | 12 | 0.06666667 |
| **16** | 4 | 0.03333333 | 15 | 0.08333333 |
| **09** | 0 | 0 | 3 | 0.01666667 |
| **10** | 2 | 0.01666667 | 1 | 0.00555556 |
| **12** | 0 | 0 | 1 | 0.00555556 |

Red: Alleles with an allele frequency < 0.05
