# Supplementary material for: HLA-DRB1*14 is associated with lower odds of symptomatic SARS-CoV-2 infection in Mexican individuals
Source: Front Immunol. 2026 Sep 1;17:1907153. doi: 10.3389/fimmu.2026.1907153 (PMC13574783; doi:10.3389/fimmu.2026.1907153)
Supplement: Supplementary file 2 [file DataSheet2.pdf]

Supplementary table S2. Allele frequencies of the population from the Metropolitan Area of the Valley of Mexico.

Table S2. Population originally from the Metropolitan Area of the Valley of Mexico. N=150

HLA-DRB1	**n**	**a.f.**
HLA-DRB1*08:02	**56**	**0.18**
HLA-DRB1*04:07	**30**	**0.1**
HLA-DRB1*14:06	**24**	**0.08**
HLA-DRB1*07:01	**19**	**0.06**
HLA-DRB1*04:04	**16**	**0.05**
HLA-DRB1*16:02	**16**	**0.05**
HLA-DRB1*14:02	**14**	**0.04**

N. Number of samples, **n.** Number of alleles, **a.f.** Allele Frequency

The *HLA-DRB1* locus was typed in symptomatic and control groups from the MAVM. Of these, the most frequent alleles were *HLA-DRB1*08:02* (a.f. 0.18), *HLA-DRB1*04:07* (a.f. 0.1), and *HLA-DRB1*14:06* (a.f. 0.08). These findings are consistent with what has been previously reported by various authors, who have documented that these alleles are highly prevalent in the Mexican population.
