# Supplementary material for: HLA-DRB1*14 is associated with lower odds of symptomatic SARS-CoV-2 infection in Mexican individuals
Source: Front Immunol. 2026 Sep 1;17:1907153. doi: 10.3389/fimmu.2026.1907153 (PMC13574783; doi:10.3389/fimmu.2026.1907153)
Supplement: Supplementary file 3 [file Table1.docx]

**Supplementary table S3**

| **Supplementary table S3. Multivariate logistic regression model evaluating the association between HLA-DRB1*14 carrier status and symptomatic SARS-CoV-2 infection** | | | | | | |
| --- | --- | --- | --- | --- | --- | --- |
| **Predictor** | **β** | **SE** | **Adjusted OR** | **95% CI** | ***p*** | **VIF** |
| ***HLA-DRB1*14*** | -1.046 | 0.397 | 0.351 | 0.161-0.765 | **0.008** | 1.02 |
| ***Age*** | 0.014 | 0.014 | 1.014 | 0.991-1.038 | **0.233** | 1.11 |
| ***BMI*** | -0.105 | 0.041 | 1.006 | 0.929-1.090 | **0.876** | 1.07 |
| ***Presence of comorbidities*** | 0.475 | 0.388 | 0.9 | 0.421-1.927 | **0.787** | 1.07 |
| ***Sex*** | 0.475 | 0.362 | 1.607 | 0.791-3.267 | **0.190** | 1.01 |
| **β.** Regression coefficient, **SE.** Standard error, **OR.** Odds ratio, **CI.** Confidence interval, ***p***. P value, **VIF.** Variance Inflation Factor. | | | | | | |

**Model-fit Information**

*The multivariable logistic regression model was fitted using data from 150 participants (90 cases and 60 controls) and included five predictors (HLA-DRB1*14 carrier status, age, sex, BMI, and presence of comorbidities), corresponding to 18 events per variable (EPV). Model fit statistics were as follows: deviance = 191, AIC = 203, BIC = 221, and McFadden's pseudo-R² = 0.059. Multicollinearity was assessed using variance inflation factors (VIF), with all values ranging from 1.01 to 1.11, indicating no evidence of problematic collinearity.*
